# A *de novo* variant of *POLR3B* causes demyelinating Charcot-Marie-Tooth disease in a Chinese patient: a case report

**DOI:** 10.1186/s12883-021-02399-y

**Published:** 2021-10-20

**Authors:** Yan-Yan Xue, Hao-Ling Cheng, Hai-Lin Dong, Hou-Min Yin, Yun Yuan, Ling-Chao Meng, Zhi-Ying Wu, Hao Yu

**Affiliations:** 1 Department of Neurology and Research Center of Neurology in Second Affiliated Hospital, Key Laboratory of Medical Neurobiology of Zhejiang Province, Zhejiang University School of Medicine, 88 Jiefang Road, Hangzhou, China; 2 Department of Neurology, First Affiliated Hospital, Fujian Medical University, Fuzhou, China; 3 Department of Neurology, Peking University First Hospital, Beijing, China

**Keywords:** *POLR3B*, Charcot-Marie-Tooth disease, Demyelinating neuropathy, *De novo*, Chinese

## Abstract

**Background:**

Charcot-Marie-Tooth (CMT) disease is a group of inherited peripheral neuropathies, which are subdivided into demyelinating and axonal forms. Biallelic mutations in *POLR3B* are the well-established cause of hypomyelinating leukodystrophy, which is characterized by hypomyelination, hypodontia, and hypogonadotropic hypogonadism. To date, only one study has reported the demyelinating peripheral neuropathy phenotype caused by heterozygous *POLR3B* variants.

**Case presentation:**

A 19-year-old male patient was referred to our hospital for progressive muscle weakness of the lower extremities. Physical examination showed muscle atrophy, sensory loss and deformities of the extremities. Nerve conduction studies and electromyography tests revealed sensorimotor demyelinating polyneuropathy with secondary axonal loss. Trio whole-exome sequencing revealed a *de novo* variant in *POLR3B* (c.3137G > A).

**Conclusions:**

In this study, we report the case of a Chinese patient with a *de novo* variant in *POLR3B* (c.3137G > A), who manifested demyelinating CMT phenotype without additional neurological or extra-neurological involvement. This work is the second report on *POLR3B*-related CMT.

## Background

Charcot-Marie-Tooth (CMT) disease is a group of inherited peripheral neuropathies clinically characterized by progressive distal muscle weakness and atrophy, sensory loss, areflexia and skeletal deformities. By virtue of electrophysiological results, it is classified into two main subgroups: demyelinating form (CMT1) and axonal form (CMT2). Biallelic mutations in *POLR3B* are the well-established cause of hypomyelinating leukodystrophy, typically featured by hypomyelination, hypodontia, and hypogonadotropic hypogonadism [1, 2]. A recent study reported six unrelated patients carrying heterozygous *de novo* variants in *POLR3B* manifested ataxia, developmental delay, spasticity and demyelinating neuropathy, which differed from previously reported phenotypes of *POLR3B*-related hypomyelinating leukodystrophy [3]. Notably, five of the six patients exhibited predominant demyelinating sensory and motor neuropathy, which unveiled for the first time a demyelinating peripheral neuropathy phenotype caused by *POLR3B* variants. However, this study has not been further validated by other studies. Here, we report a Chinese patient carrying a *de novo* variant in *POLR3B* (c.3137G > A) presenting with demyelinating CMT phenotype without other neurological or extra-neurological involvement.

## Case presentation

**Fig. 1:**
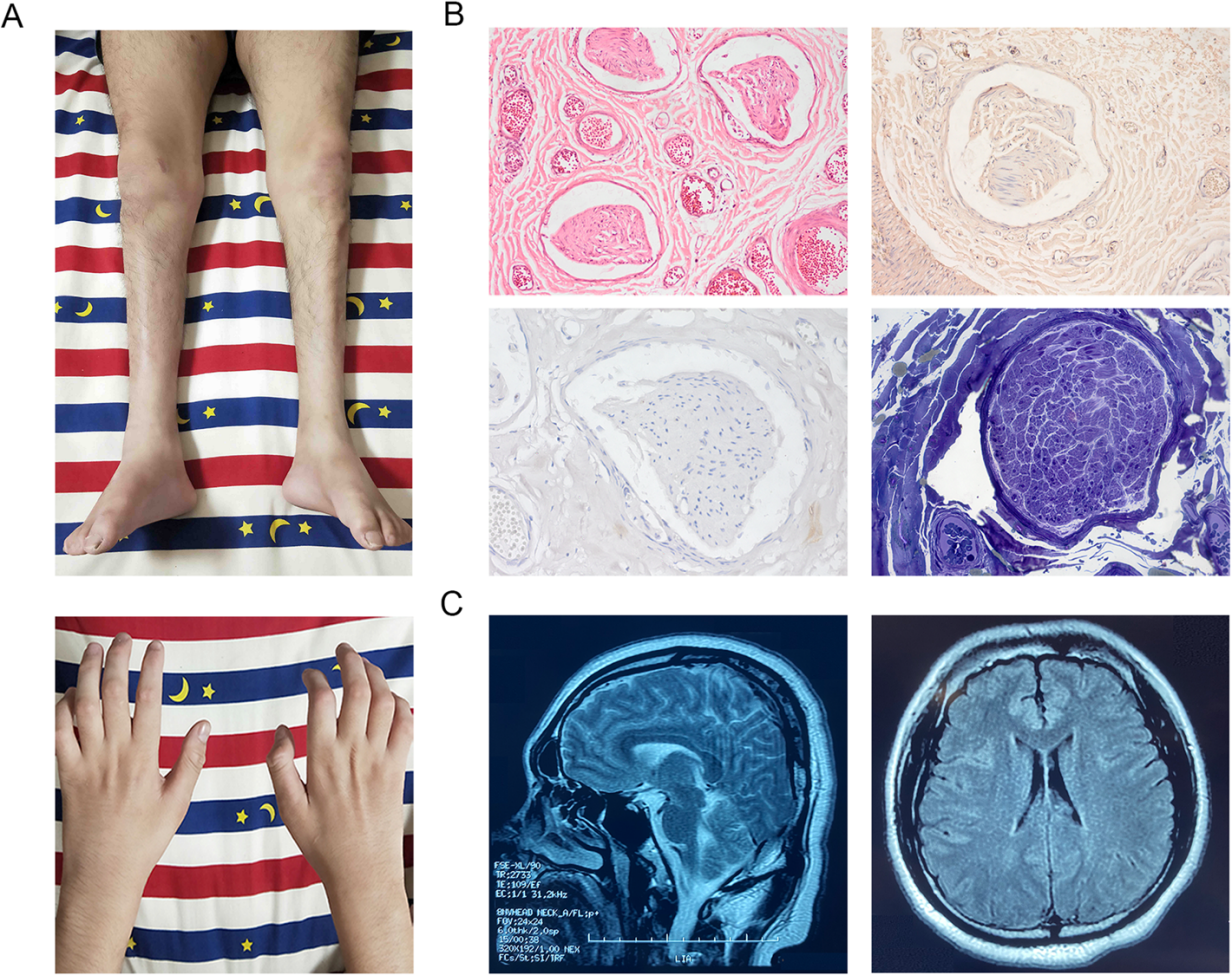
The clinical manifestations the patient. (**A**) Neurological examinations showed atrophy of the distal muscles in the extremities and pes cavus. (**B**) Sural nerve biopsy revealed a significant reduction in myelinated nerve fiber density. Hematoxylin and eosin staining (upper-left penal), Congo red staining (upper-right penal), myelin basic protein staining (lower-left penal), semithin sections (lower-right penal). (**C**) The brain MRI images of the patient

The proband was a 19-year-old man born after an uneventful pregnancy with no family history of neurologic disease. He was born at 39 weeks with a low birth weight of 2350 g. The patient started walking at around 12 months of age with no delay in achieving early developmental milestones. At 5 years old, he developed mild muscle weakness in both legs and fell frequently. Around the age of 7 years, the patient manifested symmetrical muscle atrophy of the distal lower extremities. As the condition progressed, muscle atrophy in the lower extremities worsened and extended to the upper limbs. The physical examination performed at age 8 revealed mild pes cavus and steppage gait. The bilateral knee reflexes were normal, but the ankle reflexes were absent. At 13 years old, symptoms of progressive gait deterioration and dysesthesia in the distal lower limbs were noted. At 19 years old, physical examinations revealed normal cognition, cranial nerves, and cerebellar functions, but obvious muscle atrophy in the distal muscle, pes cavus and claw hands (Fig. 1 A). Motor examination revealed 4/5 muscle strength in proximal limb muscles and wrist extensors, 3/5 in distal muscles of the lower limbs except in ankle dorsiflexors and evertors (0/5). Sensory examination showed decreased pinprick and vibration sensation in the distal extremities. Routine blood count, liver, renal and thyroid functions, blood glucose, and serum electrolyte were normal. Nerve conduction studies (NCS) and needle electromyography (EMG) tests were performed in his 8, 15 and 19 years old, and revealed progressive worsening of sensorimotor demyelinating polyneuropathy with secondary axonal loss (NCS result at the age of 8 was shown in Table 1, no response could be elicited in NCS at the age of 15 and 19). Sural nerve biopsy revealed severe loss of myelinated nerve fibers (Fig. 1B). Brain magnetic resonance imaging (MRI) revealed no specific abnormalities, including corpus callosum, cerebellum, or periventricular white matter (Fig. 1 C).

**Table 1:** Nerve conduction studies of the patient at the age of 8

Nerve	Latency	Action potential	Nerve conduction velocity
Motor nerve (left/right)	Latency (ms)	cMAP (mV)	MNCV (m/s)
Ulnar nerve	5.0/5.0	5.5/3.4	27.4/28.3
Median nerve	5.3/6.0	4.0/3.7	32.1/31/1
Peroneal nerve	NR/NR	NR/NR	NR/NR
Tibial nerve	NR/NR	NR/NR	NR/NR
Sensory nerve conduction	Latency (ms)	SNAP (µV)	SNCV (m/s)
Ulnar nerve	16.4/5.2	0.5/0.7	6.7/20.2
Median nerve	10.4/8.2	1.0/1.7	12.5/15.9
Superficial peroneal nerver	NR/NR	NR/NR	NR/NR
Sural nerve	NR/NR	NR/NR	NR/NR

Abbreviations: *cMAP* compound motor action potential; *MNCV* motor nerveconduction velocity; *NR* Non-Recordable; *SNAP* sensory nerve action potential; *SNCV* sensory nerveconduction velocity

**Fig. 2:**
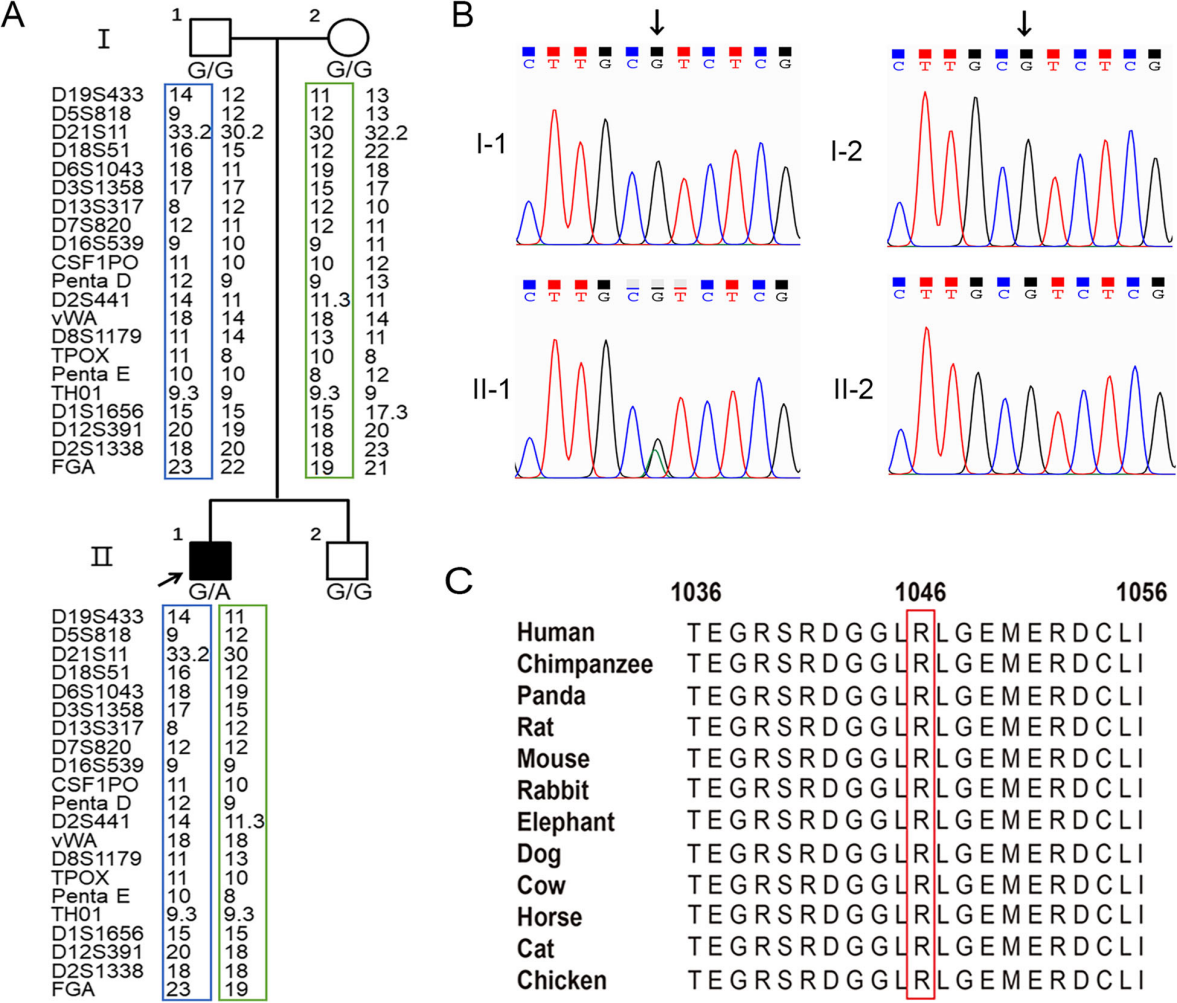
The genetic findings of the patient. (**A**) The genetic examination revealed that the patient carried a *de novo* variant (*POLR3B*: c.3137G > A, p.R1046H), and the parent-child relationship was established by parenthood analysis. (**B**) Chromatograms of the target variant. (**C**) Conservation analysis of amino acid sequences on p. R1046H variant sites are highly conserved

The patient was diagnosed with CMT based on the combination of typical progressive CMT clinical features, sural biopsy result and EMG presentation. The trio whole-exome sequencing (WES) of the proband and his parents was performed on an Illumina NovaSeq6000® in 2019, but no candidate variant of known CMT causative genes was detected at the time. The multiplex ligation-dependent probe amplification (MPLA) analysis was performed and copy number variants in *PMP22*, *MPZ* and *GJB1* were excluded. Reanalysis of the data in 2021 revealed a candidate variant in *POLR3B* gene (c.3137G > A, p.R1046H, Fig. 2 A, B**)**, which was reported early this year to be associated with a different set of clinical features including demyelinating neuropathy. The variant was *de novo*, which was confirmed by parenthood analysis (Fig. 2 A). The variant was absent from the ExAC, dbSNP, 1000G, gnomAD and our WES database which contains 1000 Chinese controls. The Arg1046 residue is highly conserved throughout vertebrate species (Fig. 2 C), suggesting that the mutation may be damaging. Also, it was predicted to be deleterious by SIFT, Polyphen-2, M-CAP, REVEL and ClinPred.

Considering hypogonadotropic hypogonadism caused by biallelic variants in *POLR3B*, serum reproductive hormone levels, including testosterone (TST), follicle stimulating hormone (FSH), luteinizing hormone (LH), estradiol (E2) and progesterone (PRG), were detected and were within normal range. Oral examination revealed no obvious abnormalities.

## Discussion and Conclusions

In our study, the first diagnosis of the patient was CMT according to his typical clinical manifestation and clinical examination results. Distal myopathies were ruled out because of severe sensory nerve involvement in the NCS and biopsy. Systemic disorders with neuropathy were ruled out because no significant central nervous system involvement or other generalized symptoms were observed and all simple blood examinations were normal [4, 5]. Early-onset pes cavus with mild sensory symptoms made the acquired demyelinating neuropathy such as CIDP unlikely. Though the clinical diagnosis was explicit, the genetic cause was not known until we reanalyzed the genetic data this year. Thus, *POLR3B* should be included in genetic testing panel for CMT. In addition, variants of uncertain significance should be routinely reevaluated considering their high reclassification rate and broader phenotypic spectrum of the corresponding genes.

Recessive mutations in *POLR3B* are recognized to cause hypomyelinating leukodystrophy. Interestingly, Djordjevic et al. have recently reported that heterozygous *de novo* variants in *POLR3B* are also pathogenic and cause a broad clinical phenotypic spectrum, including demyelinating neuropathy, seizure and spasticity [3]. In their study, one patient had the same variant (*POLR3B* c.3137G > A) and similar phenotype as our patient. Different from the other five patients in their study, the patient carrying this variant manifested predominantly demyelinating polyneuropathy without any additional neurological symptoms, such as intellectual disability, developmental delay, language difficulties, seizure and spasticity. The only difference between the two patients was that their patient showed Chiari type I malformation, which was more likely a comorbidity than a developmental consequence of the causative variant in *POLR3B.* The similar phenotypes of patients carrying the same variant (*POLR3B* c.3137G > A) may indicate a potential relationship between different genotypes and various phenotypes, which needs to be explored by further extended studies. Notably, there was no overlap among recessive and *de novo* mutations in *POLR3B*.

*POLR3B* encodes the second largest subunit of RNA polymerase III, which is involved in transcription of small non-coding RNA. Using affinity purification coupled with mass spectrometry (AP-MS), Djordjevic et al. revealed *POLR3B* c.3137G > A distinctly affected normal assembly of RNA polymerase III. Due to technical and material limitations, we did not perform functional studies on this variant, which was a limitation of our study. In addition, clinical symptoms and neurological examination results may evolve as the disease progress, and it is important to continue follow-up study.

Collectively, we report an early-onset demyelinating CMT case carrying a *de novo* variant in *POLR3B*, the second global report involving *POLR3B*-related CMT.

## Data Availability

Not applicable.

## Abbreviations

CMT: Charcot-Marie-Tooth
MRI: Magnetic resonance imaging
NCS: Nerve conduction studies
EMG: Electromyography
WES: Whole-exome sequencing
MLPA: Multiplex ligation-dependent probe amplification
